# Crystal structures of iso­quinoline–3-chloro-2-nitro­benzoic acid (1/1) and isoquinolinium 4-chloro-2-nitro­benzoate

**DOI:** 10.1107/S2056989014026152

**Published:** 2015-01-01

**Authors:** Kazuma Gotoh, Hiroyuki Ishida

**Affiliations:** a Department of Chemistry, Faculty of Science, Okayama University, Okayama 700-8530, Japan

**Keywords:** crystal structure, short hydrogen bond, chloro- and nitro-substituted benzoic acid, iso­quinoline

## Abstract

In each of the title isomeric compounds, C_9_H_7.3_N·C_7_H_3.7_ClNO_4_, (I), and C_9_H_8_N·C_7_H_3_ClNO_4_, (II), of iso­quinoline with 3-chloro-2-nitro­benzoic acid and 4-chloro-2-nitro­benzoic acid, the two components are linked by a short hydrogen bond between a base N atom and a carb­oxy O atom. In the hydrogen-bonded unit of (I), the H atom is disordered over two positions with N and O site occupancies of 0.30 (3) and 0.70 (3), respectively, while in (II), an acid–base inter­action involving H-atom transfer occurs and the H atom is located at the N site. In the crystal of (I), the acid–base units are connected through C—H⋯O hydrogen bonds into a tape structure along the *b-*axis direction. Inversion-related adjacent tapes are further linked through π–π inter­actions [centroid–centroid distances = 3.6389 (7)–3.7501 (7) Å], forming a layer parallel to (001). In the crystal of (II), the acid–base units are connected through C—H⋯O hydrogen bonds into a ladder structure along the *a-*axis direction. The ladders are further linked by another C—H⋯O hydrogen bond into a layer parallel to (001).

## Abstract 2

The structures of two isomeric compounds of iso­quinoline with 3-chloro-2-nitro­benzoic acid and 4-chloro-2-nitro­benzoic acid have been determined at 190 K. In each compound, the acid and base mol­ecules are held together by a short hydrogen bond between a carb­oxy O atom and a base N atom. In the hydrogen-bonded unit of the former, the H atom is disordered over two positions, while in the latter, an acid–base inter­action involving H-atom transfer occurs and the H atom is located at the N site.

## Chemical context   

The hydrogen bonds formed between organic acids and organic bases vary from an O—H⋯N to an O^−^⋯H—N^+^ type with increasing Δp*K*_a_ [p*K*_a_(base) − p*K*_a_(acid)], and at an appropriate Δp*K*_a_ value, a short strong hydrogen bond with a broad single minimum potential energy curve for the H atom or a double-minimum potential is observed (Jerzykiewicz *et al.*, 1998 ▸; Kalenik *et al.*, 1989 ▸; Steiner *et al.*, 2001 ▸; Schmidtmann & Wilson, 2008 ▸; Gilli & Gilli, 2009 ▸). For the system of pyridine derivative–chloro- and nitro-substituted benzoic acid (1/1), we have shown that three compounds of quinoline with 3-chloro-2-nitro­benzoic acid, 4-chloro-2-nitro­benzoic acid and 5-chloro-2-nitro­benzoic acid, and two compounds of phthal­azine with 3-chloro-2-nitro­benzoic acid and 4-chloro-2-nitro­benzoic acid have a short double-well N⋯H⋯O hydrogen bond between the aromatic N atom and the carb­oxy O atom (Gotoh & Ishida, 2009 ▸, 2011*a* ▸).
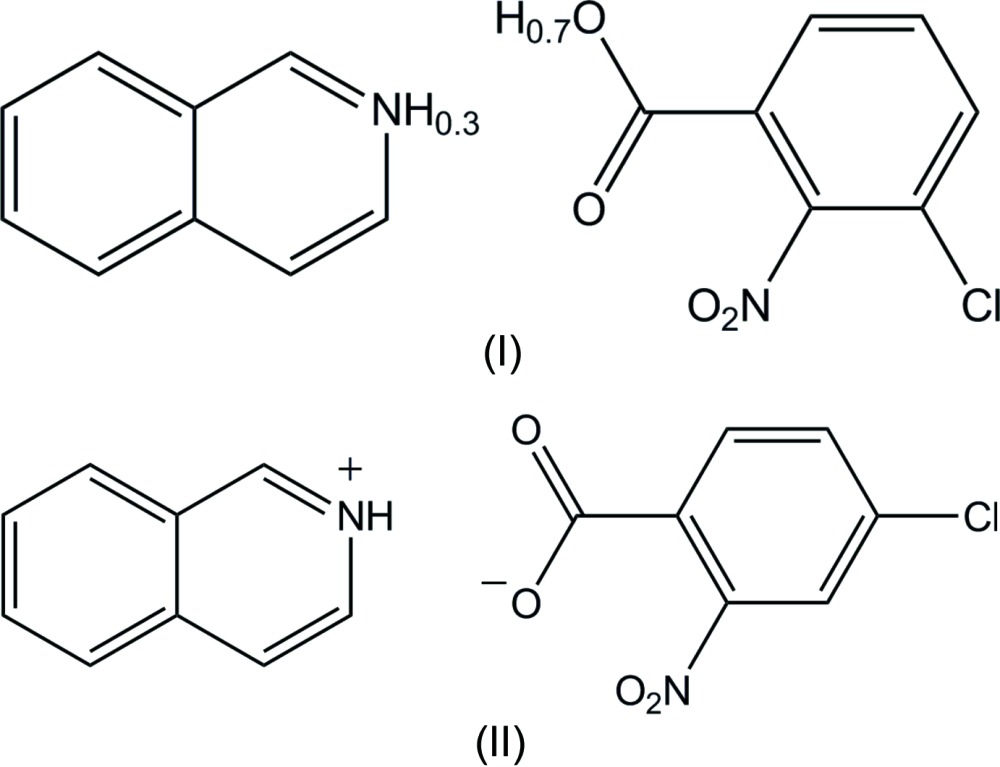


We report here two isomeric compounds of iso­quinoline with chloro- and nitro-substituted benzoic acids, namely, iso­quinoline–3-chloro-2-nitro­benzoic acid (1/1), (I), and 4-chloro-2-nitro­benzoate isoquinolinium, (II), in order to extend our studies of hydrogen bonding in the system of pyridine derivative–chloro- and nitro-substituted benzoic acid (Gotoh & Ishida, 2011*b* ▸,*c* ▸).

## Structural commentary   

**Figure 1:**
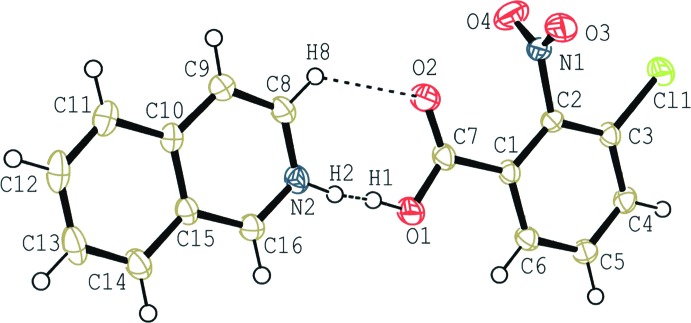
A mol­ecular view of (I), showing the atom-numbering scheme. Displacement ellipsoids are drawn at the 50% probability level and H atoms are shown as small spheres of arbitrary radii. The disordered O—H⋯N/N—H⋯O hydrogen bond and the C—H⋯O inter­action are indicated by dashed lines.

The mol­ecular structure of (I) is shown in Fig. 1 ▸. The base and acid mol­ecules are held together by a short hydrogen bond between the N atom of the base and the carb­oxy O atom. The H atom in the hydrogen bond is disordered over two positions with the N and O sites occupancies refined to 0.30 (3) and 0.70 (3), respectively. In addition, a C—H⋯O hydrogen bond (C8—H8⋯O2; Table 1 ▸) is observed in the hydrogen-bonded acid–base unit. In the unit, the iso­quinoline ring system, the carb­oxy group and the benzene ring of the acid mol­ecule are almost coplanar with each other; the carb­oxy group makes dihedral angles of 5.35 (15) and 5.91 (15)°, respectively, with the iso­quinoline ring system and the benzene ring, and the dihedral angle between the iso­quinoline ring system and the benzene ring is 1.21 (4)°. On the other hand, the nitro group and the benzene ring are almost perpendicular with a dihedral angle of 83.71 (13)°.

**Figure 2:**
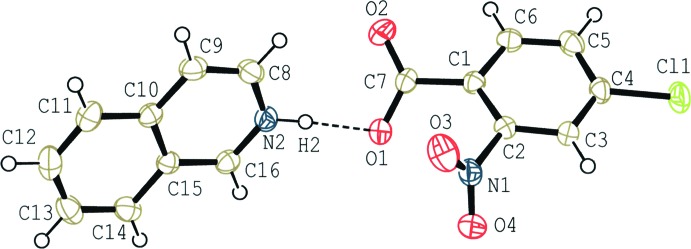
A mol­ecular view of (II), showing the atom-numbering scheme. Displacement ellipsoids are drawn at the 50% probability level and H atoms are shown as small spheres of arbitrary radii.

The mol­ecular structure of (II) is shown in Fig. 2 ▸. An acid–base inter­action involving H-atom transfer occurs and the base and acid mol­ecules are linked by an N^+^—H⋯O^−^ hydrogen bond. In the hydrogen-bonded unit, the iso­quinoline ring system make dihedral angles of 54.12 (15) and 71.89 (5)°, respectively, with the carb­oxy group and the benzene ring of the acid. In the acid mol­ecule, the benzene ring makes dihedral angles of 26.59 (15) and 67.69 (15)°, respectively, with the carb­oxy and nitro groups.

## Supra­molecular features   

**Figure 3:**
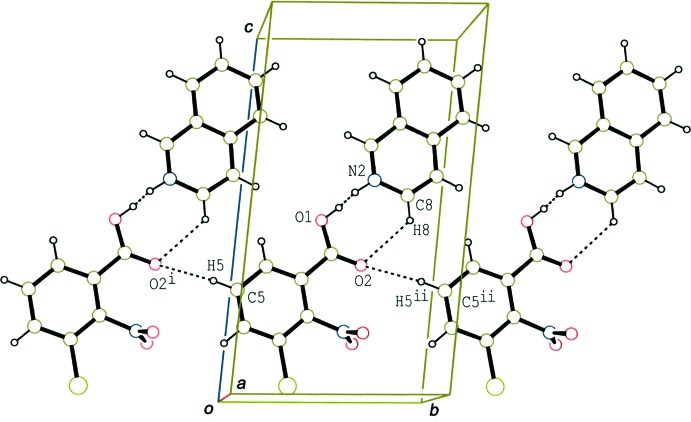
A packing diagram of (I), showing the hydrogen-bonded tape structure along the *b* axis. The dashed lines indicate disordered O—H⋯N/N—H⋯O hydrogen bonds and the C—H⋯O inter­actions. [Symmetry codes: (i) *x*, *y* − 1, *z*; (ii) *x*, *y* + 1, *z*.]

**Figure 4:**
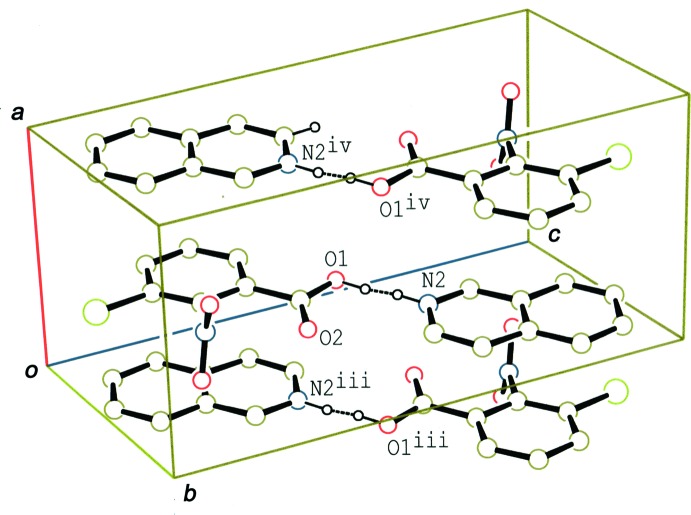
A packing diagram of (I), showing the π–π stacking structure along the *a* axis. The dashed lines indicate disordered O—H⋯N/N—H⋯O hydrogen bonds and H atoms not involved in the hydrogen bonds have been omitted. [Symmetry codes: (iii) −*x*, −*y* + 1, −*z* + 1; (iv) −*x* + 1, −*y* + 1, −*z* + 1.]

In the crystal of (I), the hydrogen-bonded acid-base units are linked by a C—H⋯O hydrogen bond (C5—H5⋯O2^i^; Table 1 ▸), forming a tape structure along the *b-*axis direction (Fig. 3 ▸). Adjacent tapes, which are related by an inversion center, are further linked through π–π inter­actions between the benzene ring of the acid and the iso­quinoline ring system (Fig. 4 ▸), forming a layer parallel to the (001) plane. The centroid–centroid distances are in the range 3.6389 (7)–3.7501 (7) Å [*Cg*1⋯*Cg*2^iii^ = 3.7501 (7), *Cg*1⋯*Cg*2^iv^ = 3.6674 (7), *Cg*1⋯*Cg*3^iii^ = 3.6637 (7) and *Cg*1⋯*Cg*3^iv^ = 3.6389 (7) Å, where *Cg*1, *Cg*2 and *Cg*3 are the centroids of the C1–C6 benzene ring of the acid, and the N2/C8–C10/C15/C16 rings of the base, respectively. Symmetry codes: (iii) −*x*, −*y* + 1, −*z* + 1; (iv) −*x* + 1, −*y* + 1, −*z* + 1.]

**Figure 5:**
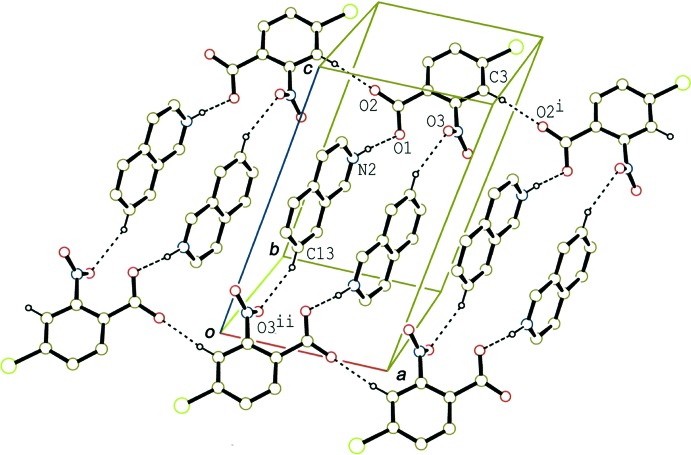
A packing diagram of (II), showing the hydrogen-bonded ladder structure along the *a* axis. The dashed lines indicate N—H⋯O and C—H⋯O hydrogen bonds. H atoms not involved in the hydrogen bonds have been omitted. [Symmetry codes: (i) *x* + 1, *y*, *z*; (ii) −*x* + 1, −*y*, −*z* + 1.]

In the crystal of (II), the acid–base units are connected through C—H⋯O hydrogen bonds (C3—H3⋯O2^i^ and C13—H13⋯O3^ii^; Table 2 ▸) into a ladder structure along the *a-*axis direction (Fig. 5 ▸). Adjacent ladders are further linked by another C—H⋯O hydrogen bond (C16—H16⋯O1^iii^; Table 2 ▸), forming a layer parallel to the (001) plane.

## Database survey   

A search of the Cambridge Structural Database (Version 5.35, last update May 2014; Groom & Allen, 2014 ▸) showed 49 structures of co-crystals/salts of pyridine (or amine) derivative–chloro- and nitro-substituted benzoic acid: 16 structures containing 2-chloro-4-nitro­benzoic acid, nine for 2-chloro-5-nitro­benzoic acid, three for 3-chloro-2-nitro­benzoic acid, five for 3-chloro-6-nitro­benzoic acid, eight for 4-chloro-2-nitro­benzoic acid and eight for 4-chloro-3-nitro­benzoic acid. On the other hand, there were eight structures of co-crystals/salts of iso­quinoline with organic acids. The N⋯O distances of the N—H⋯O/O—H⋯N hydrogen bonds are in the range 2.578 (2)–2.8718 (17) Å. No disordered H atoms were observed in the hydrogen bonds.

## Synthesis and crystallization   

Crystals of compounds (I) and (II) were obtained by slow evaporation from aceto­nitrile solutions of iso­quinoline with the corresponding chloro- and nitro-substituted benzoic acid in a 1:1 molar ratio at room temperature [50 ml aceto­nitrile solution of iso­quinoline (0.202 g) and 3-chloro-2-nitro­benzoic acid (0.315 g) for (I), and 150 ml solution of iso­quinoline (0.204 g) and 4-chloro-2-nitro­benzoic acid (0.318 g) for (II)].

## Refinement   

Crystal data, data collection and structure refinement details are summarized in Table 3 ▸. All H atoms in compounds (I) and (II) were found in difference Fourier maps. The H atom in (I), which is involved in the N⋯H⋯O hydrogen bonds, was found to be disordered over two positions in a difference Fourier map. Since the site-occupancy factors and isotropic displacement parameters were strongly correlated, the occupancy factors were refined, with *U*_iso_(H) = 1.5*U*_eq_(N or O). The positional parameters were refined with bond restraints of O—H = 0.84 (2) Å and N—H = 0.88 (2) Å. Atom H2 in (II) was refined freely [refined distance N2—H2 = 0.91 (2) Å]. Other H atoms of compounds (I) and (II) were positioned geometrically (C—H = 0.95 Å) and treated as riding, with *U*_iso_(H) = 1.2*U*_eq_(C).

## Supplementary Material

Crystal structure: contains datablock(s) General, I, II. DOI: 10.1107/S2056989014026152/lh5740sup1.cif

Structure factors: contains datablock(s) II. DOI: 10.1107/S2056989014026152/lh5740IIsup3.hkl

CCDC references: 1036583, 1036582

Additional supporting information:  crystallographic information; 3D view; checkCIF report

## Figures and Tables

**Table 1:** Hydrogen-bond geometry (, ) for (I)

*D*H*A*	*D*H	H*A*	*D* *A*	*D*H*A*
O1H1N2	0.84(2)	1.74(2)	2.5725(12)	177(2)
N2H2O1	0.88(2)	1.69(5)	2.5725(12)	172(5)
C5H5O2^i^	0.95	2.49	3.3427(14)	149
C8H8O2	0.95	2.53	3.1977(14)	128

Symmetry code: (i) 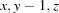.

**Table 2:** Hydrogen-bond geometry (, ) for (II)

*D*H*A*	*D*H	H*A*	*D* *A*	*D*H*A*
N2H2O1	0.91(2)	1.67(2)	2.5738(14)	169(2)
C3H3O2^i^	0.95	2.21	3.1580(15)	174
C13H13O3^ii^	0.95	2.52	3.3405(19)	145
C16H16O1^iii^	0.95	2.43	3.3477(15)	163

Symmetry codes: (i) 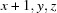; (ii) 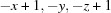; (iii) 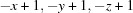.

**Table 3:** Experimental details

	(I)	(II)
Crystal data		
Chemical formula	C_9_H_7.3_NC_7_H_3.7_ClNO_4_	C_9_H_8_N^+^C_7_H_3_ClNO_4_
*M*_r_	330.73	330.73
Crystal system, space group	Triclinic, *P* 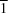	Triclinic, *P* 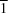
Temperature (K)	190	190
*a*, *b*, *c* ()	6.93986(15), 7.6629(5), 13.9475(5)	7.5916(3), 7.7607(3), 13.0456(4)
, , ()	83.945(3), 87.6039(16), 85.117(4)	74.8360(11), 80.1736(10), 80.3642(13)
*V* (^3^)	734.50(6)	724.84(4)
*Z*	2	2
Radiation type	Mo *K*	Mo *K*
(mm^1^)	0.28	0.29
Crystal size (mm)	0.35 0.28 0.10	0.39 0.32 0.23

Data collection		
Diffractometer	Rigaku R-AXIS RAPIDII	Rigaku R-AXIS RAPIDII
Absorption correction	Numerical (*NUMABS*; Higashi, 1999 ▸)	Numerical (*NUMABS*; Higashi, 1999 ▸)
*T*_min_, *T*_max_	0.918, 0.972	0.903, 0.936
No. of measured, independent and observed [*I* > 2(*I*)] reflections	15432, 4278, 3729	21510, 4224, 3559
*R*_int_	0.022	0.024
(sin /)_max_ (^1^)	0.704	0.704

Refinement		
*R*[*F* ^2^ > 2(*F* ^2^)], *wR*(*F* ^2^), *S*	0.034, 0.098, 1.08	0.039, 0.117, 1.07
No. of reflections	4278	4224
No. of parameters	219	212
No. of restraints	2	0
H-atom treatment	H atoms treated by a mixture of independent and constrained refinement	H atoms treated by a mixture of independent and constrained refinement
_max_, _min_ (e ^3^)	0.41, 0.22	0.42, 0.16

Computer programs: *PROCESS-AUTO* (Rigaku/MSC, 2004 ▸), *SHELXS97* and *SHELXL2014* (Sheldrick, 2008 ▸), *ORTEP-3 for Windows* (Farrugia, 2012 ▸), *CrystalStructure* (Rigaku, 2010 ▸) and *PLATON* (Spek, 2009 ▸).

## supplementary crystallographic information

### Crystal data

**Table:**

C_9_H_8_N^+^·C_7_H_3_ClNO_4_^−^	*Z* = 2
*M**_r_* = 330.73	*F*(000) = 340.00
Triclinic, *P*1	*D*_x_ = 1.515 Mg m^−^^3^
Hall symbol: -P 1	Mo *K*α radiation, λ = 0.71075 Å
*a* = 7.5916 (3) Å	Cell parameters from 17960 reflections
*b* = 7.7607 (3) Å	θ = 3.0–30.1°
*c* = 13.0456 (4) Å	µ = 0.29 mm^−^^1^
α = 74.8360 (11)°	*T* = 190 K
β = 80.1736 (10)°	Block, colorless
γ = 80.3642 (13)°	0.39 × 0.32 × 0.23 mm
*V* = 724.84 (4) Å^3^	

### Data collection

**Table:**

Rigaku R-AXIS RAPIDII diffractometer	3559 reflections with *I* > 2σ(*I*)
Detector resolution: 10.000 pixels mm^-1^	*R*_int_ = 0.024
ω scans	θ_max_ = 30.0°
Absorption correction: numerical (*NUMABS*; Higashi, 1999)	*h* = −10→10
*T*_min_ = 0.903, *T*_max_ = 0.936	*k* = −10→10
21510 measured reflections	*l* = −18→18
4224 independent reflections	

### Refinement

**Table:**

Refinement on *F*^2^	0 restraints
Least-squares matrix: full	Hydrogen site location: mixed
*R*[*F*^2^ > 2σ(*F*^2^)] = 0.039	H atoms treated by a mixture of independent and constrained refinement
*wR*(*F*^2^) = 0.117	*w* = 1/[σ^2^(*F*_o_^2^) + (0.0699*P*)^2^ + 0.126*P*] where *P* = (*F*_o_^2^ + 2*F*_c_^2^)/3
*S* = 1.07	(Δ/σ)_max_ = 0.001
4224 reflections	Δρ_max_ = 0.42 e Å^−^^3^
212 parameters	Δρ_min_ = −0.16 e Å^−^^3^

### Special details

**Table:**

Geometry. All e.s.d.'s (except the e.s.d. in the dihedral angle between two l.s. planes) are estimated using the full covariance matrix. The cell e.s.d.'s are taken into account individually in the estimation of e.s.d.'s in distances, angles and torsion angles; correlations between e.s.d.'s in cell parameters are only used when they are defined by crystal symmetry. An approximate (isotropic) treatment of cell e.s.d.'s is used for estimating e.s.d.'s involving l.s. planes.
Refinement. Reflections were merged by *SHELXL* according to the crystal class for the calculation of statistics and refinement.

### Fractional atomic coordinates and isotropic or equivalent isotropic displacement parameters (Å^2^)

**Table:**

	*x*	*y*	*z*	*U*_iso_*/*U*_eq_	
Cl1	0.89304 (5)	0.83272 (5)	0.98941 (3)	0.04513 (12)	
O1	0.49156 (12)	0.48077 (13)	0.69271 (7)	0.0359 (2)	
O2	0.25649 (12)	0.50987 (15)	0.81971 (8)	0.0427 (2)	
O3	0.78736 (17)	0.25408 (13)	0.80657 (10)	0.0503 (3)	
O4	0.91034 (13)	0.46177 (16)	0.68317 (9)	0.0465 (3)	
N1	0.81282 (13)	0.40954 (14)	0.76667 (9)	0.0328 (2)	
N2	0.31990 (13)	0.26809 (14)	0.63693 (8)	0.0313 (2)	
C1	0.53627 (14)	0.58384 (14)	0.83982 (8)	0.0253 (2)	
C2	0.72336 (14)	0.54307 (14)	0.82731 (8)	0.0257 (2)	
C3	0.83671 (15)	0.61582 (16)	0.87214 (9)	0.0293 (2)	
H3	0.9639	0.5862	0.8608	0.035*	
C4	0.75517 (17)	0.73430 (15)	0.93451 (9)	0.0310 (2)	
C5	0.56930 (18)	0.77436 (16)	0.95367 (10)	0.0338 (2)	
H5	0.5166	0.8522	0.9992	0.041*	
C6	0.46123 (16)	0.69972 (15)	0.90582 (9)	0.0299 (2)	
H6	0.3339	0.7281	0.9182	0.036*	
C7	0.41584 (15)	0.51806 (15)	0.78102 (9)	0.0281 (2)	
C8	0.24974 (17)	0.14032 (19)	0.71997 (10)	0.0357 (3)	
H8	0.2368	0.1551	0.7910	0.043*	
C9	0.19825 (16)	−0.00741 (18)	0.70253 (10)	0.0344 (2)	
H9	0.1497	−0.0957	0.7610	0.041*	
C10	0.21705 (14)	−0.02954 (15)	0.59708 (9)	0.0282 (2)	
C11	0.17073 (17)	−0.18170 (17)	0.57248 (12)	0.0363 (3)	
H11	0.1257	−0.2761	0.6282	0.044*	
C12	0.19101 (18)	−0.19246 (19)	0.46842 (13)	0.0417 (3)	
H12	0.1586	−0.2946	0.4524	0.050*	
C13	0.25903 (18)	−0.0555 (2)	0.38392 (11)	0.0404 (3)	
H13	0.2719	−0.0664	0.3121	0.048*	
C14	0.30629 (16)	0.09202 (18)	0.40472 (9)	0.0339 (2)	
H14	0.3528	0.1839	0.3478	0.041*	
C15	0.28566 (14)	0.10747 (15)	0.51186 (9)	0.0264 (2)	
C16	0.33645 (15)	0.25579 (16)	0.53682 (9)	0.0294 (2)	
H16	0.3834	0.3486	0.4808	0.035*	
H2	0.369 (3)	0.354 (3)	0.6545 (17)	0.060 (6)*	

### Atomic displacement parameters (Å^2^)

**Table:**

	*U*^11^	*U*^22^	*U*^33^	*U*^12^	*U*^13^	*U*^23^
Cl1	0.0583 (2)	0.0493 (2)	0.03860 (18)	−0.02822 (16)	−0.00558 (14)	−0.01675 (14)
O1	0.0312 (4)	0.0486 (5)	0.0341 (4)	−0.0119 (4)	−0.0042 (3)	−0.0166 (4)
O2	0.0260 (4)	0.0626 (6)	0.0428 (5)	−0.0125 (4)	−0.0022 (3)	−0.0155 (4)
O3	0.0648 (7)	0.0320 (5)	0.0575 (6)	0.0025 (4)	−0.0139 (5)	−0.0187 (4)
O4	0.0342 (5)	0.0670 (7)	0.0459 (5)	−0.0106 (4)	0.0058 (4)	−0.0311 (5)
N1	0.0273 (4)	0.0377 (5)	0.0386 (5)	0.0004 (4)	−0.0087 (4)	−0.0185 (4)
N2	0.0251 (4)	0.0361 (5)	0.0365 (5)	−0.0036 (4)	−0.0051 (4)	−0.0146 (4)
C1	0.0255 (5)	0.0250 (5)	0.0250 (5)	−0.0044 (4)	−0.0037 (4)	−0.0044 (4)
C2	0.0260 (5)	0.0265 (5)	0.0258 (5)	−0.0040 (4)	−0.0029 (4)	−0.0085 (4)
C3	0.0278 (5)	0.0340 (5)	0.0286 (5)	−0.0085 (4)	−0.0039 (4)	−0.0090 (4)
C4	0.0404 (6)	0.0285 (5)	0.0281 (5)	−0.0131 (4)	−0.0055 (4)	−0.0078 (4)
C5	0.0436 (6)	0.0274 (5)	0.0317 (5)	−0.0042 (4)	−0.0024 (5)	−0.0114 (4)
C6	0.0295 (5)	0.0287 (5)	0.0298 (5)	−0.0013 (4)	−0.0021 (4)	−0.0070 (4)
C7	0.0257 (5)	0.0289 (5)	0.0304 (5)	−0.0061 (4)	−0.0070 (4)	−0.0045 (4)
C8	0.0319 (6)	0.0482 (7)	0.0287 (5)	−0.0040 (5)	−0.0029 (4)	−0.0133 (5)
C9	0.0311 (5)	0.0417 (6)	0.0274 (5)	−0.0074 (5)	−0.0014 (4)	−0.0028 (4)
C10	0.0220 (5)	0.0308 (5)	0.0309 (5)	−0.0021 (4)	−0.0054 (4)	−0.0052 (4)
C11	0.0296 (5)	0.0312 (5)	0.0491 (7)	−0.0039 (4)	−0.0098 (5)	−0.0082 (5)
C12	0.0349 (6)	0.0391 (6)	0.0602 (8)	0.0020 (5)	−0.0175 (6)	−0.0247 (6)
C13	0.0360 (6)	0.0527 (8)	0.0381 (6)	0.0052 (5)	−0.0128 (5)	−0.0227 (6)
C14	0.0298 (5)	0.0430 (6)	0.0278 (5)	0.0002 (5)	−0.0062 (4)	−0.0080 (4)
C15	0.0207 (4)	0.0313 (5)	0.0267 (5)	−0.0012 (4)	−0.0048 (4)	−0.0063 (4)
C16	0.0228 (5)	0.0328 (5)	0.0323 (5)	−0.0035 (4)	−0.0044 (4)	−0.0068 (4)

### Geometric parameters (Å, º)

**Table:**

Cl1—C4	1.7326 (12)	C6—H6	0.9500
O1—C7	1.2784 (15)	C8—C9	1.3550 (19)
O2—C7	1.2340 (14)	C8—H8	0.9500
O3—N1	1.2162 (15)	C9—C10	1.4108 (17)
O4—N1	1.2220 (15)	C9—H9	0.9500
N1—C2	1.4734 (14)	C10—C11	1.4131 (17)
N2—C16	1.3176 (16)	C10—C15	1.4168 (15)
N2—C8	1.3632 (17)	C11—C12	1.363 (2)
N2—H2	0.91 (2)	C11—H11	0.9500
C1—C6	1.3931 (15)	C12—C13	1.410 (2)
C1—C2	1.3933 (15)	C12—H12	0.9500
C1—C7	1.5118 (15)	C13—C14	1.3590 (19)
C2—C3	1.3814 (15)	C13—H13	0.9500
C3—C4	1.3855 (16)	C14—C15	1.4140 (16)
C3—H3	0.9500	C14—H14	0.9500
C4—C5	1.3869 (18)	C15—C16	1.4025 (16)
C5—C6	1.3862 (17)	C16—H16	0.9500
C5—H5	0.9500		
			
O3—N1—O4	125.41 (11)	C9—C8—N2	120.89 (11)
O3—N1—C2	116.47 (10)	C9—C8—H8	119.6
O4—N1—C2	118.08 (10)	N2—C8—H8	119.6
C16—N2—C8	121.77 (11)	C8—C9—C10	119.69 (11)
C16—N2—H2	121.1 (13)	C8—C9—H9	120.2
C8—N2—H2	116.6 (13)	C10—C9—H9	120.2
C6—C1—C2	116.80 (10)	C9—C10—C11	123.14 (11)
C6—C1—C7	119.68 (10)	C9—C10—C15	118.36 (11)
C2—C1—C7	123.42 (10)	C11—C10—C15	118.50 (11)
C3—C2—C1	124.21 (10)	C12—C11—C10	119.70 (12)
C3—C2—N1	115.37 (9)	C12—C11—H11	120.2
C1—C2—N1	120.38 (9)	C10—C11—H11	120.2
C2—C3—C4	116.52 (10)	C11—C12—C13	121.55 (12)
C2—C3—H3	121.7	C11—C12—H12	119.2
C4—C3—H3	121.7	C13—C12—H12	119.2
C3—C4—C5	121.97 (10)	C14—C13—C12	120.33 (12)
C3—C4—Cl1	117.91 (9)	C14—C13—H13	119.8
C5—C4—Cl1	120.12 (9)	C12—C13—H13	119.8
C6—C5—C4	119.35 (11)	C13—C14—C15	119.39 (12)
C6—C5—H5	120.3	C13—C14—H14	120.3
C4—C5—H5	120.3	C15—C14—H14	120.3
C5—C6—C1	121.06 (11)	C16—C15—C14	121.11 (11)
C5—C6—H6	119.5	C16—C15—C10	118.34 (10)
C1—C6—H6	119.5	C14—C15—C10	120.53 (11)
O2—C7—O1	126.47 (11)	N2—C16—C15	120.90 (11)
O2—C7—C1	118.47 (11)	N2—C16—H16	119.6
O1—C7—C1	115.02 (9)	C15—C16—H16	119.6
			
C6—C1—C2—C3	−2.85 (16)	C2—C1—C7—O1	−24.68 (15)
C7—C1—C2—C3	173.44 (10)	C16—N2—C8—C9	1.66 (18)
C6—C1—C2—N1	174.65 (10)	N2—C8—C9—C10	−0.10 (19)
C7—C1—C2—N1	−9.05 (16)	C8—C9—C10—C11	178.39 (11)
O3—N1—C2—C3	110.00 (12)	C8—C9—C10—C15	−1.62 (17)
O4—N1—C2—C3	−67.61 (14)	C9—C10—C11—C12	179.31 (11)
O3—N1—C2—C1	−67.71 (14)	C15—C10—C11—C12	−0.68 (17)
O4—N1—C2—C1	114.67 (12)	C10—C11—C12—C13	0.57 (19)
C1—C2—C3—C4	1.06 (17)	C11—C12—C13—C14	−0.04 (19)
N1—C2—C3—C4	−176.56 (10)	C12—C13—C14—C15	−0.36 (19)
C2—C3—C4—C5	1.76 (17)	C13—C14—C15—C16	178.64 (11)
C2—C3—C4—Cl1	−178.43 (8)	C13—C14—C15—C10	0.24 (17)
C3—C4—C5—C6	−2.62 (18)	C9—C10—C15—C16	1.85 (15)
Cl1—C4—C5—C6	177.57 (9)	C11—C10—C15—C16	−178.16 (10)
C4—C5—C6—C1	0.69 (18)	C9—C10—C15—C14	−179.71 (10)
C2—C1—C6—C5	1.91 (16)	C11—C10—C15—C14	0.28 (16)
C7—C1—C6—C5	−174.53 (10)	C8—N2—C16—C15	−1.40 (17)
C6—C1—C7—O2	−26.60 (16)	C14—C15—C16—N2	−178.81 (10)
C2—C1—C7—O2	157.21 (11)	C10—C15—C16—N2	−0.38 (16)
C6—C1—C7—O1	151.51 (11)		

### Hydrogen-bond geometry (Å, º)

**Table:**

*D*—H···*A*	*D*—H	H···*A*	*D*···*A*	*D*—H···*A*
N2—H2···O1	0.91 (2)	1.67 (2)	2.5738 (14)	169 (2)
C3—H3···O2^i^	0.95	2.21	3.1580 (15)	174
C13—H13···O3^ii^	0.95	2.52	3.3405 (19)	145
C16—H16···O1^iii^	0.95	2.43	3.3477 (15)	163

Symmetry codes: (i) *x*+1, *y*, *z*; (ii) −*x*+1, −*y*, −*z*+1; (iii) −*x*+1, −*y*+1, −*z*+1.
